# Assessment of CORA-based levelling osteotomy in the feline stifle: an *ex vivo* limb press study

**DOI:** 10.1007/s11259-026-11361-7

**Published:** 2026-06-18

**Authors:** Parisa Mazdarani, James Edward Miles

**Affiliations:** 1 Department of Veterinary Clinical Sciences, University of Copenhagen, Dyrlaegevej 16, Frederiksberg C, 1870 Denmark; 2 Department of Small Animal Clinical Sciences, Cornell University, Ithaca, NY USA

**Keywords:** Feline stifle biomechanics, Cranial tibial translation, *Ex vivo* limb modelling, Muscle force simulation, Tibial plateau modification

## Abstract

Cranial cruciate ligament (CCL) rupture is uncommon in cats but may cause persistent stifle instability. Previous ex vivo assessments of tibial plateau levelling osteotomy and tibial tuberosity advancement have failed to restore stability in the feline stifle. This study evaluated whether centre-of-rotation-of-angulation–based levelling osteotomy (CBLO) improves ex vivo stability in a refined feline limb-press model that preserves hip flexion–extension. Ten feline hindlimbs were tested intact, after CCL transection, and after CBLO under 30% bodyweight axial loading. Radiographic measurements included proxy CCL distance, joint angles, tibial plateau angle (TPA), anatomical–mechanical axis (AMA) angle, and gastrocnemius lever-arm characteristics. Muscle forces were recorded using calibrated load cells. CBLO reduced mean TPA from 23.8° (SD 2.2°) to 9.4° (SD 2.5°). AMA angle after CBLO (0.5°, SD 0.9°) did not differ significantly from zero (95% CI -0.1° to 1.1°, *P* = 0.1). Joint condition significantly affected proxy CCL distance (*P* < 0.001). Compared to intact limbs, distances increased by 7.1 mm (95% CI 6.0 to 8.2, *P* < 0.001) after CCL transection and by 6.1 mm (95% CI 4.9 to 7.3, *P* < 0.001) after CBLO, with no significant difference between CCL-deficient and CBLO limbs (-1.0 mm; 95% CI -2.6 to 0.5, *P* = 0.3). Joint angles and simulated muscle forces were unaffected by joint condition. Gastrocnemius lever-arm length using the calcaneal tunnel was 86% (SD 2.0%) of the anatomical length. CBLO, as performed in this model, corrected tibial slope but did not restore ex vivo stability of the feline stifle following CCL transection.

**Supplementary Information:**

The online version contains supplementary material available at https://doi.org/10.1007/s11259-026-11361-7.

## Introduction

Cranial cruciate ligament (CCL) rupture in cats is less common than in dogs and is often trauma-associated (McLaughlin 2002; Harasen 2005), but several osteotomy techniques have been applied clinically with apparent success (Hoots and Petersen 2005; Perry and Fitzpatrick 2010; Allan 2014; Mindner et al. 2016; Bula and Perry 2021). Previous ex vivo feline studies of tibial plateau levelling osteotomy (Bilmont et al. 2018) and tibial tuberosity advancement (Retournard et al. 2016) have consistently failed to restore stability, in contrast to results from a meta-analysis of canine limb-press studies (Chaves et al. 2025). Potential reasons include species differences in tibial plateau topologies, restriction of muscle simulation to quadriceps and gastrocnemius (ignoring the soleus), and poor construct validity (Mazdarani et al. 2025). Reported quadriceps forces in earlier feline limb models (Koch et al. 2021) were markedly lower than in vivo patellar ligament loads (Hasler et al. 1997), which may have reduced the sensitivity of those models to detect stabilizing effects.

Recent work refining the feline limb-press model by preserving hip flexion–extension has yielded quadriceps muscle force magnitudes and quadriceps: gastrocnemius ratios closer to those measured in walking cats, potentially improving construct validity (Mazdarani et al. 2025).

The centre of rotation of angulation–based levelling osteotomy (CBLO) is intended to neutralize cranial tibial thrust by treating tibial plateau slope as an angular deformity (Raske et al. 2013). Although biomechanical (Mazdarani et al. 2022) and clinical work (Raske et al. 2013; Coskun and Viskjer 2023) in dogs suggests potential benefits, CBLO has not been evaluated in the cat.

Based on the principles of CBLO and improved construct validity of the refined limb press model, we hypothesized that CBLO would reduce tibial plateau slope and thereby restore ex vivo craniocaudal stability of the feline stifle.

## Methods

Hindlimbs from ten skeletally mature feline cadavers, euthanised for reasons unrelated to this study and donated for research, were obtained following institutional approval (2022-16). Limbs showing skeletal immaturity, joint instability or previous orthopaedic trauma were excluded.

### Limb-press model and mounting

**Fig. 1:**
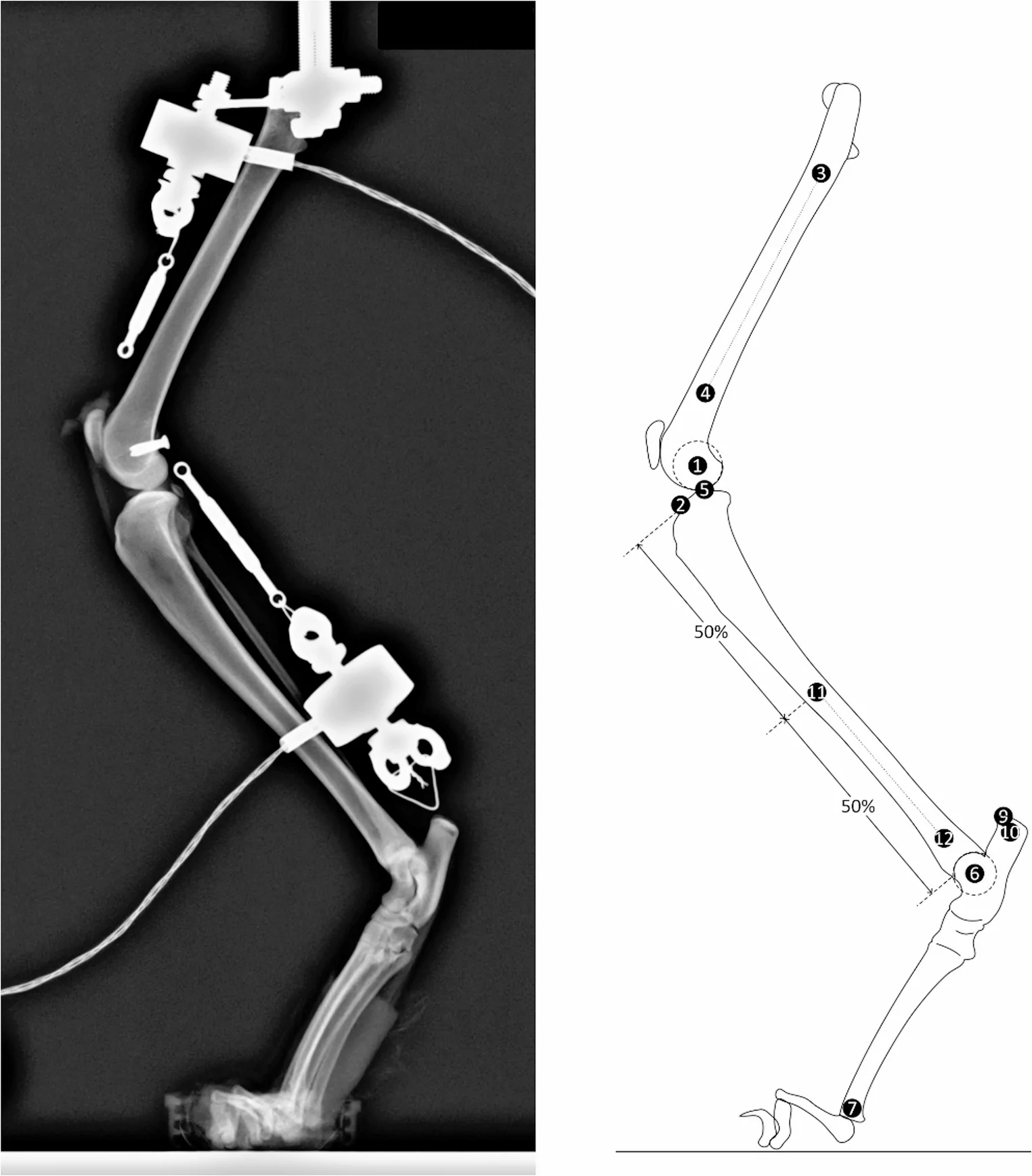
Limb-press setup used for *ex vivo* testing. Radiograph and corresponding line drawing of a feline hindlimb positioned in the limb press. The proximal femur is supported by a mediolateral pin secured within external fixator clamps, while the foot is supported in a receiving cup mounted on a digital scale to monitor axial load. Simulated quadriceps and gastrocnemius forces are applied using calibrated load cells and turnbuckles. Points 1–2 indicate the measurement landmarks used as a proxy for cranial cruciate ligament length. Points 3–4 (femur) with 5–6 (tibia) define the stifle angle, and points 5–6 together with point 7 define the hock angle. Points 9–10 identify the anatomical attachment of the common calcaneal tendon and the location of the calcaneal bone tunnel. Points 11–12 represent the distal tibial axis used for osteotomy planning

A refined limb-press model retaining hip flexion–extension was used, as previously described (Mazdarani et al. 2025; Mazdarani and Miles 2026), to generate simulated quadriceps and gastrocnemius forces approaching physiologic ratios in cats (Fig. 1). The proximal femur was supported using a mediolateral pin and external fixator clamps, and the foot rested in a receiving cup mounted on a digital scale, ensuring consistent foot location throughout each testing series. Stifle and hock joint angles were set to 120° ± 5°, and femoral angle to 60° ± 2°, following prior recommendations for feline limb-press testing (Retournard et al. 2016; Bilmont et al. 2018; Koch et al. 2021). Simulated quadriceps and gastrocnemius forces were applied using turnbuckles in series with DYMH-103 calibrated load cells (0–10 kg range; safe overload 150% rated capacity). Load cells were connected via HX711 signal amplifiers (SparkFun, Niwot, CO, USA) to a DRFduino UNO R3 microcontroller board (DFRobot, Shanghai, China). Signals were calibrated and monitored using publicly available routines as previously described (Mazdarani et al. 2025), with calibration performed at the start and end of each experimental day and monitored throughout testing. Detailed load cell specifications are provided in Supplementary Table 1. An axial load equivalent to 30% bodyweight was applied once acceptable positioning had been confirmed radiographically.

### Joint preparation and CCL transection

After baseline measurements, the cranial cruciate ligament (CCL) was transected under direct visualization via a limited medial arthrotomy. Complete section was confirmed via cranial drawer testing, and the arthrotomy was closed using simple interrupted sutures. Limb positioning was re-established by readjusting femoral, stifle, and hock joint angles to the predefined target values, with alignment confirmed radiographically, prior to repeated axial loading.

### CBLO procedure

**Fig. 2:**
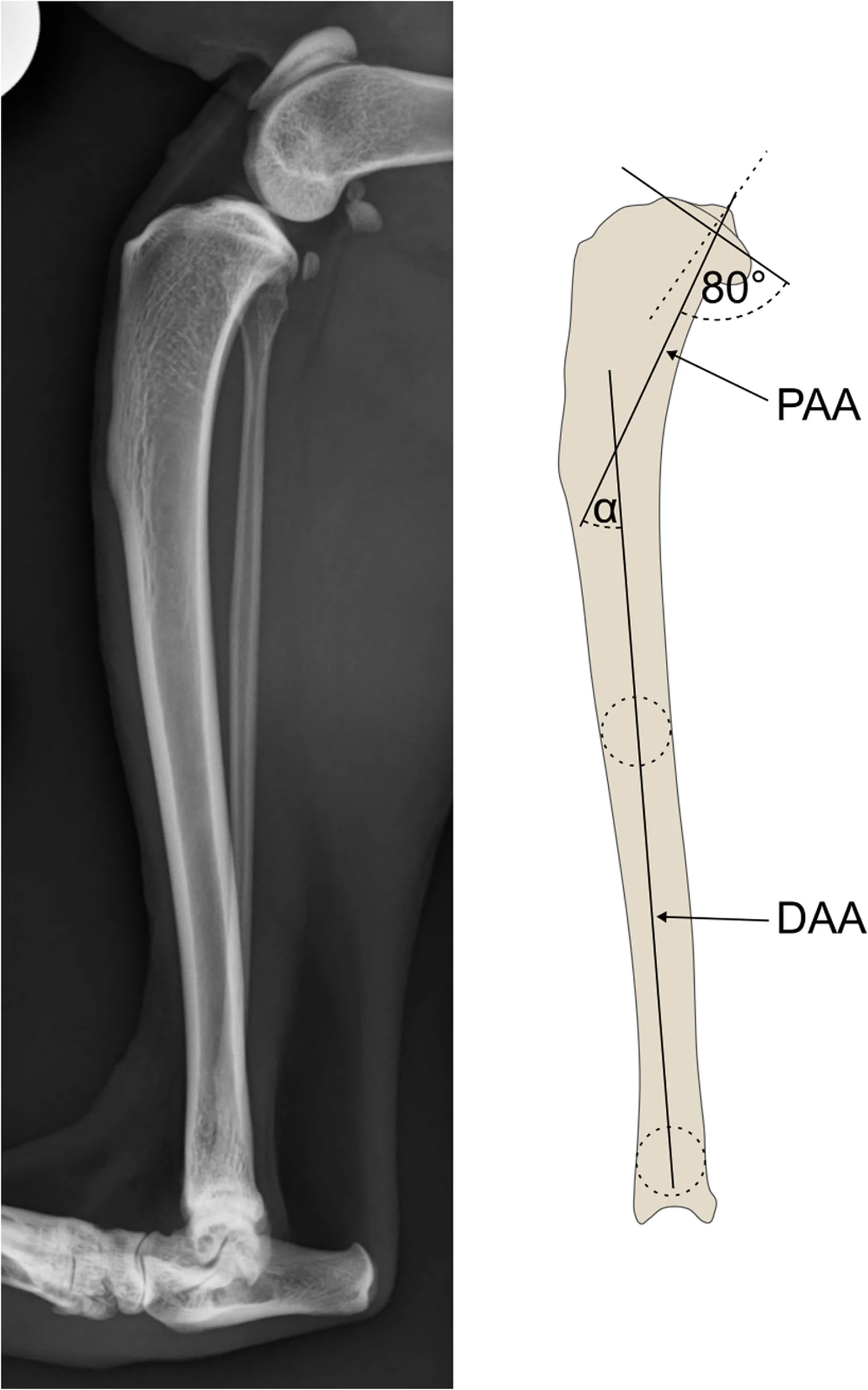
Planning of the CORA-based levelling osteotomy (CBLO). Mediolateral radiograph and corresponding line drawing of a feline tibia. The proximal anatomical axis (PAA) is offset from the tibial plateau line by 80° for a target tibial plateau angle of 10°. The distal anatomical axis (DAA) passes through diaphyseal centres at 50% of the tibial length and distally. The intersection of PAA and DAA locates the centre of rotation of angulation (CORA), and the acute angle (α) between the two represents the correction angle. Centring an osteotomy at the CORA and rotating the proximal segment cranially by α aligns the PAA and DAA with the mechanical axis, reducing the tibial plateau angle to the predefined target

The limb was then dismounted for CBLO. CORA locations and correction angles were determined from radiographs using established methods (Raske et al. 2013) with a target TPA of 10° (Fig. 2). A radial osteotomy was performed using a 12-mm TPLO blade, and the proximal segment rotated cranially according to the measured correction angle, before stabilization with a 1.6-mm Kirschner wire and a 2-mm dynamic compression plate. The limb was repositioned in the press; alignment was confirmed radiographically and loading repeated.

### Radiographic measurements

Femoral and joint angles, tibial plateau angle, anatomical–mechanical axis angle, proxy CCL distance (femorotibial distance), and lever-arm characteristics for the simulated muscles were measured using Fiji software (Schindelin et al. 2012) (Fig. 1). Measurement methods followed those previously described for feline limb-press studies (Koch et al. 2021; Mazdarani and Miles 2026). Acceptable images required femoral condylar superimposition within 1 mm (Kneifel et al. 2018; Koch et al. 2021).

### Data analysis

Simulated quadriceps and gastrocnemius forces were normalized to bodyweight, and quadriceps: gastrocnemius ratios calculated. Normality was assessed using Shapiro–Wilk tests, and repeated-measures ANOVA with Bonferroni-corrected pairwise comparisons was used to evaluate effects of joint condition (intact, CCL-deficient, CBLO) on outcome variables. Effect sizes were calculated using partial omega-squared, with cut-offs between small, medium and large effects at 0.13 and 0.26. Lever arm length was compared using a paired *t*-test on intact joint measurements, and the post-CBLO anatomical–mechanical axis (AMA) angle was assessed using a one-sample *t*-test against the target value of 0°.

Statistical significance was set at *P* < 0.05.

## Results

All data and residuals were normally distributed. Mean body mass was 3.8 kg (SD 1.0 kg). Initial tibial plateau angle (TPA) was 23.8° (SD 2.2°) and was reduced to 9.4° (SD 2.5°) after CBLO. The mean correction angle was 21.4° (SD 4.1°). The CORA was located a mean of 35.1 mm (SD 6.4 mm) from the tibial plateau and 27.4 mm (SD 6.5 mm) from the tibial tuberosity. The mid-tibial plateau was 12.4 mm (SD 2.3 mm) caudal to the distal tibial axis initially, reducing to 1.0 mm (SD 2.2 mm) post-CBLO. Mean initial anatomical–mechanical axis angle was 6.4° (SD 1.0°). After CBLO, the mean angle was 0.5° (SD 0.9°), which did not differ significantly from zero (*P* = 0.10; 95% CI: − 0.1° to 1.1°). Key outcome variables are summarized in Table 1.

**Table 1:** Femorotibial distance, angles, and forces. The femorotibial distance serves as a proxy for cranial tibial translation. Femur angle was measured relative to the horizontal: stifle and hock angles represent caudal and cranial angles, respectively. Raw simulated quadriceps and gastrocnemius forces along with body-weight-normalized values and their ratio are reported

	Intact	CCLx	CBLO
Femorotibial distance (mm)	9.7 (1.7)	16.8 (2.0)	15.8 (2.8)
Femur angle (°)	61.2 (1.4)	60.7 (1.0)	61.2 (0.8)
Stifle angle (°)	118.7 (1.9)	120.7 (2.2)	119.6 (3.0)
Hock angle (°)	119.3 (2.3)	118.6 (3.6)	119.1 (2.3)
Quadriceps force (N)	47.4 (11.8)	45.0 (14.3)	49.9 (18.0)
Gastrocnemius force (N)	23.7 (5.8)	24.6 (11.1)	26.3 (12.3)
Force ratio (Q/G)	2.01 (0.31)	1.93 (0.43)	1.95 (0.26)
Normalized quadriceps force	1.29 (0.16)	1.22 (0.21)	1.34 (0.22)
Normalized gastrocnemius force	0.65 (0.09)	0.65 (0.13)	0.7 (0.14)

*CCLx *cranial cruciate ligament transected, *CBLO *CORA-based levelling osteotomy

Joint condition significantly affected the proxy CCL distance (F_(2,18)_ = 150.2, *P* < 0.001, $\:{\omega\:}_{p}^{2}$ = 0.968). Mean distance increased from intact to CCL-deficient by 7.1 mm (95% CI: 6.0 to 8.2, *P* < 0.001) and from intact to CBLO by 6.1 mm (95% CI: 4.9 to 7.3, *P* < 0.001). The difference between CCL-deficient and CBLO was not significant at -1.0 mm (95% CI: -2.6 to 0.5, *P* = 0.3).

Joint angles did not significantly differ between joint statuses for either femoral (F_(2,18)_ = 0.48, *P* = 0.63, $\:{\omega\:}_{p}^{2}\:$= -0.117), stifle joint (F_(2,18)_ = 1.47, *P* = 0.26, $\:{\omega\:}_{p}^{2}$ = 0.086) or hock joint (F_(2,18)_ = 0.18, *P* = 0.84, $\:{\omega\:}_{p}^{2}$ = -0.197) angles. Similarly, muscle force ratio (F_(2,18)_ = 0.16, *P* = 0.86, $\:{\omega\:}_{p}^{2}\:$= -0.204), normalized quadriceps force (F_(2,18)_ = 1.75, *P* = 0.20, $\:{\omega\:}_{p}^{2}$ = 0.136) and normalized gastrocnemius force (F_(2,18)_ = 0.94, *P* = 0.41, $\:{\omega\:}_{p}^{2}$ = 0) did not differ significantly with joint status.

Gastrocnemius lever arm length using the bone tunnel was 86% (SD 2.0%) compared to the insertion of the common calcaneal tendon, with talus-tunnel and talus-calcaneus lengths of 14.2 mm (SD 2.0 mm) and 16.3 mm (SD 1.9 mm), respectively. Talus-tunnel lengths were significantly lower (95% CI: -1.8 to -2.4, *P* < 0.001), potentially increasing the required gastrocnemius force in this model.

## Discussion

In this refined feline limb-press model, CBLO did not restore ex vivo stifle stability following CCL transection. Proxy CCL distance remained significantly increased in both CCL-deficient and CBLO conditions, with no meaningful difference between them, indicating persistent cranial tibial translation despite angular correction. These findings contrast with canine ex vivo studies where CBLO or similar osteotomies frequently improve stability but align with previous feline TPLO and TTA limb-press studies that also failed to demonstrate stabilization (Retournard et al. 2016; Bilmont et al. 2018).

Although target TPA was reduced appropriately, minor long-axis shifts and unavoidable imprecision in ACA–CORA alignment may also have contributed to residual instability. Whether alternative target angles or modifications of surgical technique would yield better results remains unknown. However, even over-reduction of the proximal segment in a feline TPLO model failed to produce stability (Bilmont et al. 2018). It is also possible that CBLO, irrespective of testing conditions, may not reliably stabilize the feline stifle in vivo, reflecting species specific biomechanical differences rather than limitations of experimental models alone.

Several limitations of the experimental model should be considered when interpreting these findings. First, simulated muscle loads in feline limb-press models, although improved with hip mobility, may still under-represent physiologic quadriceps–gastrocnemius ratios, potentially diminishing any stabilizing effect of CBLO. Second, the use of a calcaneal bone tunnel shortened the gastrocnemius lever arm, increasing the force required to counter tibiotarsal joint flexion. Alternative fixation methods which maintain lever arm length should be explored. Third, species-specific differences in hindlimb musculature may also influence stability. Cats possess a functional soleus muscle, which contributes to tarsal extension and normally shares the plantarflexion load with the gastrocnemius (Hudson and Hamilton 2010). Its absence in the model places the entire extensor demand on the gastrocnemius, potentially amplifying the impact of the shortened gastrocnemius lever arm. Fourth, differences in tibial plateau morphology may limit the transferability of canine CBLO rationale to cats. Fifth, a recent canine study (Coskun and Viskjer 2023) documented a cranially-oriented distal axis unlike that originally described and used here, but still achieved a mean post-CBLO TPA of 10.8°. The significance of axis choice remains unclear. Finally, as with all cadaveric limb press models, the absence of neuromuscular control, dynamic stabilization, and biological adaptation limits direct clinical extrapolation. Furthermore, the use of static axial loading equivalent to 30% body weight may underestimate physiologic or peak in vivo forces, potentially masking stabilization effects that might emerge under different loading conditions.

Rotational stability was not assessed in this study; given the failure to restore craniocaudal stability following CBLO, rotational measurements were considered unlikely to alter the primary interpretation, but this remains a limitation of the current biomechanical evaluation.

The limited sample size may increase the risk of type II error for smaller biomechanical effects, particularly for secondary outcomes that showed no significant differences.

Overall, these findings suggest that CBLO in this feline model does not confer measurable ex vivo stability in the feline stifle, reinforcing concerns that feline limb-press models may require further refinement or additional muscle group simulation to accurately evaluate osteotomy-based stabilization techniques, and that optimal CBLO parameters for cats remain undefined. Model optimization and species-specific biomechanical considerations should be explored before extrapolating canine osteotomy strategies to cats.

## Supplementary Information

Below is the link to the electronic supplementary material.

Supplementary Material 1

## Data Availability

The datasets generated during and/or analysed during the current study are available in the Figshare repository, [https://doi.org/10.6084/m9.figshare.31841719].
