# Supplementary material for: Assessment of CORA-based levelling osteotomy in the feline stifle: an ex vivo limb press study
Source: Vet Res Commun. 2026 Jun 18;50(5):402. doi: 10.1007/s11259-026-11361-7 (PMC13279282; doi:10.1007/s11259-026-11361-7)
Supplement: Supplementary file 1 — Supplementary Material 1 [file 11259_2026_11361_MOESM1_ESM.pdf]

Supplementary Information

Article title: *Assessment of CORA-based levelling osteotomy in the feline stifle: an ex vivo limb press study*

Journal: *Veterinary Research Communications*

Authors: Parisa Mazdarani, James Edward Miles

Corresponding author: James Edward Miles, Department of Veterinary Clinical Sciences, University of Copenhagen, Dyrlægevej 16, 1870 Frederiksberg C, Denmark, Email: jami@sund.ku.dk

Supplementary Table 1: Technical specifications of the DYMH-103 load cells used for force measurement in the present study. Specifications are provided to document measurement performance of the experimental setup.

Parameter	Specification
Range	0-10 kg
Output sensitivity	1.0-1.5±10% mV/V
Zero output	±2% full scale
Nonlinear	0.3% full scale
Lag	0.03% full scale
Repeatability	0.03% full scale
Creep (30 minutes)	0.2% full scale
Temperature sensitivity drift	0.1% full scale/10 °C
Zero temperature drift	0.1% full scale/10 °C
Material	Stainless steel
Resistance	350 Ω
Insulation resistance	≥5000 MΩ/100 V DC
Use voltage	0-10 V
Operating temperature range	-20~80 °C
Safe overload	150%
Extreme overload	200%
Cable connection	Ex+: red; Ex-: black; Sig+: green; Sig-: white
